# A case report of ceftriaxone-induced cardiopulmonary arrest

**DOI:** 10.1016/j.amsu.2022.104813

**Published:** 2022-11-05

**Authors:** Faith Abodunrin, Mahmoud Ismayl, Ahmed Aboeata, Robert Plambeck

**Affiliations:** a Department of Medicine, Creighton University School of Medicine, Omaha, NE, USA; b Department of Medicine, Division of Cardiology, Creighton University School of Medicine, Omaha, NE, USA; c Department of Medicine, Division of Pulmonary/Critical Care, Creighton University School of Medicine, Omaha, NE, USA

**Keywords:** Ceftriaxone, Cardiac arrest, Asystole, Case report

## Abstract

**Introduction:**

and Importance: Ceftriaxone is used frequently in treating infectious diseases. While hypersensitivity skin reactions are common with the use of ceftriaxone, anaphylactic reactions are rare.

**Case presentation:**

A 66-year-old female presented to our hospital with complaints of headache and sinus congestion. Vital signs showed hypoxia, and the physical exam was unremarkable. A computed tomography scan of the chest revealed right upper lobe pneumonia, and the patient was started on ceftriaxone and azithromycin. The patient went into asystole 1 min after ceftriaxone administration. She did not require cardiopulmonary resuscitative measures as she spontaneously transitioned to normal sinus rhythm. Given the timing of the event immediately after ceftriaxone administration, we determined ceftriaxone was the likely culprit. The patient received alternative treatment for pneumonia and recovered without sequelae. We added ceftriaxone to her allergy list.

**Clinical discussion:**

This case report highlights a rare adverse event associated with ceftriaxone. After an extensive literature search, we found only four other reported cases of cardiopulmonary arrest following ceftriaxone. The exact mechanism for this adverse event has not been fully elucidated.

**Conclusion:**

Clinicians should be aware of the potential for ceftriaxone-induced asystole, perform allergy reviews and obtain informed consent before its administration.

## Highlights

• Anaphylactic reactions are rare with the use of ceftriaxone.
• Only 4 reported cases of cardiac arrest following ceftriaxone have been published.
• Clinicians should be aware of the potential risk for ceftriaxone-induced asystole.
• Clinicians should review allergies and obtain informed consent prior to ceftriaxone administration.

## 1 Introduction and Importance

Ceftriaxone is a commonly used third-generation cephalosporin indicated for the management of different bacterial infections. It has the longest half-life of all cephalosporins and is administered parenterally. Ceftriaxone has a good safety profile making it a frequent drug of choice in treating infections. However, certain adverse reactions have been reported with its use [1,2]. Hypersensitivity dermatologic reactions occur with a frequency of 1–3% with the use of ceftriaxone [1,2]. Ceftriaxone-related anaphylaxis occurs between 0.001 and 0.1% [2]. Little is known about cardiovascular events associated with ceftriaxone. We report a rare case of a 66-year-old female patient who went into asystole after a single dose of intravenous ceftriaxone. To our knowledge, this is the 5th documented case of ceftriaxone-induced cardiopulmonary arrest. The work has been reported in line with the SCARE 2020 Criteria [3].

## 2 Case Presentation

A 66-year-old female with a past medical history significant for type 2 diabetes mellitus and hypertension presented to the hospital with complaints of shortness of breath, sinus congestion, and cough. She denied any chest pain, palpitations, or wheezing. In addition, there was no pertinent surgical history or family history. She denied the use of alcohol, tobacco, or illicit drugs. She also had no known drug allergies. Vital signs were as follows: blood pressure 177/84 mmHg, heart rate 92 beats per minute, and temperature 36.4 C. She was hypoxic and was placed on 2 L of supplemental oxygen via nasal cannula. On physical examination, the patient was alert and conscious but appeared to be in mild respiratory distress. Breath sounds were diffusely decreased bilaterally.

**Fig. 1:**
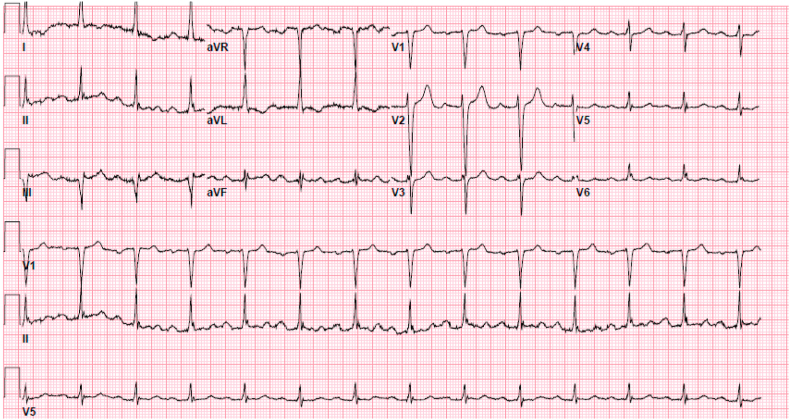
Electrocardiography showing normal sinus rhythm with 1st-degree atrioventricular block.

**Fig. 2:**
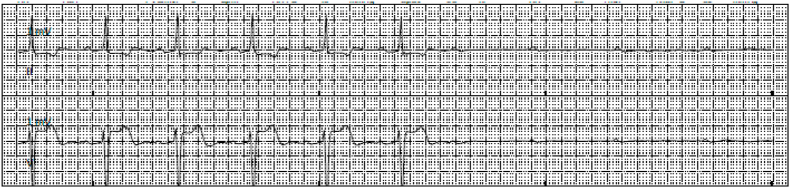
Telemetry strip showing normal sinus rhythm followed by asystole rhythm.

**Fig. 3:**
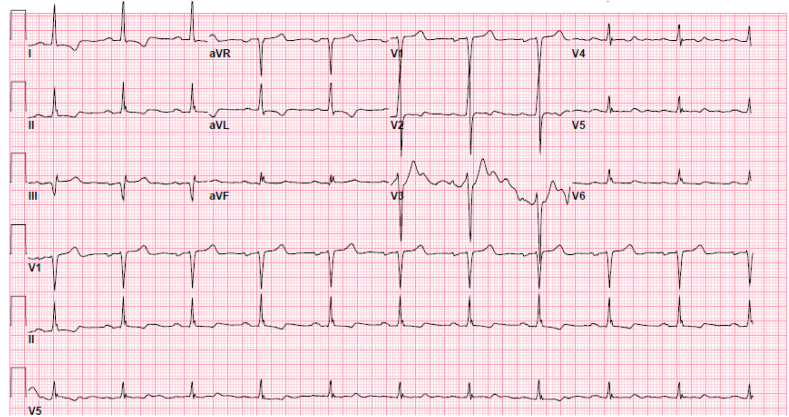
Electrocardiography showing normal sinus rhythm and no ischemic changes.

The initial electrocardiogram on day 1 showed normal sinus rhythm with a 1st-degree atrioventricular block (Fig. 1). Relevant laboratory tests revealed normal white blood cell count 10.1 k/uL, hemoglobin 14.5 mg/dL, platelets 380k/uL, blood urea nitrogen 18 mg/dl, and creatinine 0.9 mg/dl. Procalcitonin was elevated at 0.59 ng/mL, indicative of possible bacterial infection. A computed tomography scan of the chest showed right upper lobe pneumonia. The patient was started on ceftriaxone and azithromycin to treat community-acquired pneumonia (CAP). One minute after administering 1g of intravenous ceftriaxone, the patient went into asystole as seen on telemetry (Fig. 2). The event lasted for 30 seconds, after which the patient returned to normal sinus rhythm as seen on the electrocardiogram (Fig. 3) without requiring chest compressions. She was lethargic after the event and was intubated for airway protection. Alternative treatment for pneumonia was initiated and ceftriaxone was added to her list of allergies. The patient's clinical status improved and she was extubated the next day. No other cardiac events were reported on telemetry monitoring throughout her hospitalization. The patient was safely discharged on day 4 of hospitalization on oral antibiotics with a close follow-up with her primary care provider in 1 week.

## 3 Clinical Discussion

Adverse drug reactions account for 4–30% of hospital admissions in the United States and Canada, 6–19% in Australia, and 3–11% in Europe [4]. Signs and symptoms of anaphylaxis can be unpredictable and variable, ranging from mild skin rash and generalized edema to fatal reactions such as bronchoconstriction and hypotension [5]. Therefore, the absence of one or more of the common symptoms of anaphylaxis does not rule it out [6]. Mortality associated with anaphylaxis usually occurs due to respiratory or cardiovascular failure, or both [6].

Anaphylaxis to ceftriaxone is quite rare [2]. Lin et al. reported 17 cases of ceftriaxone-related anaphylaxis [7]. However, only 4 case reports on ceftriaxone-induced cardiopulmonary arrest have been published worldwide [[8], [9], [10], [11]]. In a case by Riezzo et al., ceftriaxone-related cardiopulmonary arrest occurred in the setting of an intradermal skin test with ceftriaxone, after which cardiopulmonary arrest occurred [8]. The cause of death was listed as fatal anaphylactic shock caused by ceftriaxone intradermal injection [8]. Notably, a detailed informed patient consent form had not been received in that case, and the judge ruled that the physician was negligent in the test procedures and with respect to his delay in managing the anaphylactic reaction [7]. In another case by Saritas et al., a 55-year-old man who presented to the emergency department (ED) with high-grade fever, abdominal pain, dysuria, and weakness experienced asystole 1 min after his first dose of ceftriaxone [9]. Cardiopulmonary resuscitation and tracheal intubation were performed immediately, and the ceftriaxone infusion was discontinued. Circulation was restored within 20 minutes with no short-term sequelae [9]. In another case by Ul Mustafa et al., a 9-year-old girl presenting with sore throat and a small abscess on the cheek experienced cardiopulmonary arrest within a minute of intravenous ceftriaxone administration [10]. That patient expired despite approximately 20 minutes of cardiopulmonary resuscitation in the ED. Finally, in a case by Aboul-Fotouh et al., grade IV perianesthetic anaphylaxis occurred in a 44-year-old man after a single injected dose of ceftriaxone was introduced for surgical prophylaxis leading to severe bronchospasm, hypotension, bradycardia, cardiac arrest (asystole), and respiratory arrest [11]. The patient was successfully resuscitated and regained consciousness 2 hours later. It is important to note that our patient was alert and conscious before the intravenous administration of ceftriaxone and had cardiopulmonary arrest within a minute after receiving ceftriaxone. This observation was similar to the case described by Ul Mustafa et al. [10]. The patient did not show typical signs and symptoms of anaphylaxis before the cardiopulmonary arrest [10]. Published literature on ceftriaxone-induced cardiopulmonary arrest [[8], [9], [10], [11]] is shown in Table 1.

**Table 1:** Published data related to ceftriaxone-induced cardiopulmonary arrest.

Authors	Age (years), gender	Reason for administration	Mode of administration	Fatality
Riezzo et al. [8]	59, Male	Intradermal skin test with ceftriaxone	Intradermal	Yes
Saritas et al. [9]	55, Male	Bacterial infection	Intravenous	No
Ul Mustafa et al. [10]	9, Female	Sore throat and small abscess on cheek	Intravenous	Yes
Aboul-Fotouh et al. [11]	44, Male	Surgical prophylaxis	Intravenous	No

## 4 Conclusions

This case highlights the importance of obtaining a proper drug allergy history and informed patient consent before administering ceftriaxone. Physicians should be aware of the risk of anaphylaxis and asystole that may occur after the first dose of ceftriaxone and be ready to manage it properly.

## Provenance and peer review

Not commissioned, externally peer-reviewed.

## Ethical approval

Given the nature of the article, a case report, no ethical approval was required.

## Sources of funding for your research

This research did not receive any specific grant from funding agencies in the public, commercial, or not-for-profit sectors.

## Author contribution

All authors contributed to this manuscript.

Faith Abodunrin: Writing - original draft.

Mahmoud Ismayl: Writing - original draft.

Ahmed Aboeata: Supervision; reviewing and editing.

Robert Plambeck: Supervision; reviewing and editing.

## Registration of research studies

This is not an original research project involving human participants in an interventional or an observational study but a case report. This registration was not required.

## Guarantor

Faith Abodunrin, MD faithabodunrin@creighton.edu.

## Consent

Written informed consent was obtained from the patient for publication of this case report and accompanying images. A copy of the written consent is available for review by the Editor-in-Chief of this journal on request.

## Declaration of competing interest

The authors have no conflict of interest to declare.
